# Silver(I) complexes with phenolic Schiff bases: Synthesis, antibacterial evaluation and interaction with biomolecules

**DOI:** 10.5599/admet.1167

**Published:** 2022-09-13

**Authors:** Natalia Loginova, Maxim Gvozdev, Nikolai Osipovich, Alina Khodosovskaya, Tatiana Koval’chuk-Rabchinskaya, Galina Ksendzova, Dzmitry Kotsikau, Anatoly Evtushenkov

**Affiliations:** 1 Faculty of Chemistry, Belarusian State University, Leningradskaya str. 14, 220030 Minsk, Belarus; 2 Research Institute for Physico-Chemical Problems of the Belarusian State University, Leningradskaya str. 14, 220030 Minsk, Belarus; 3 Faculty of Biology, Belarusian State University, 4 Independence Avenue, 220030 Minsk, Belarus

**Keywords:** Silver, Phenolic compounds, Nanoparticles, Antimicrobial activity, BSA binding, Cytochrome *c* reduction

## Abstract

Novel Ag(I) complexes (**2a**–**2c**) with phenolic Schiff bases were synthesized using 4,6-di-tert-butyl-3-(((5-mercapto-1,3,4-thiadiazol-2-yl)imino)methyl)benzene-1,2-diol (**1a**), 4,6-di-tert-butyl-3-(((4-mercaptophenyl)imino)methyl)benzene-1,2-diol (**1b**), and 4,6-di-tert-butyl-3-(((3-mercaptophenyl)imino)methyl)benzene-1,2-diol (**1c**). They were examined by elemental analysis, FT-IR, UV-Vis, ^1^H-NMR spectroscopy, XRD, cyclic voltammetry, conductivity measurements, and biological methods. The complexes are characterized by distorted geometry of the coordination cores AgN_2_S_2_ (**2c**), AgNS (**2b**) and AgS_2_ (**2a**). These stable complexes were not typified by the intramolecular redox reaction in organic solvents resulting in the formation of silver nanoparticles (AgNPs). Antibacterial activity of **1a**–**1c** and **2a**–**2c** was evaluated in comparison with AgNPs and commonly used antibiotics. All the complexes were more active than the ligands against the bacteria tested (14), but they were less active than AgNPs and commonly used antibiotics. Both **1a**–**1c** and their complexes **2a**–**2c** exhibited the capability for the bovine heart Fe(III)-Cyt c reduction. The ligands **1b** and **1c** were characterized by the highest reduction rate among the compounds under study, and they showed a higher reducing ability (determined by cyclic voltammetry) as compared with that of their Ag(I) complexes **2b** and **2c**.

## Introduction

The development of novel effective antibacterial agents assumes prime importance in the context of the ever-growing frequency of infections caused by the strains of bacteria resistant to widely used pharmaceuticals [1]. Silver is biocidal in its ionic form and, unlike many antibiotics, has several various mechanisms of antimicrobial action. In particular, sulphhydryl groups both in bacterial cell walls and enzymes were shown to be vulnerable to denaturation; nucleoproteins and nucleic acids are additional target sites [2]. These multiple targets render the appropriate mutations required for microorganisms to become resistant unlikely. Moreover, silver-containing pharmaceuticals have the following advantages: a low induction of resistance compared to antibiotics; a broad spectrum of activities (bacteria, yeasts and moulds); and safety in a proper dose [2]. In this connection, silver-containing pharmaceuticals turned out to be promising for medicine again.

Pharmacological screening of the Ag(I) complexes with biologically active substances for antimicrobial activity represents a strategy for the development of a novel class of antimicrobials that have a different mode of action compared to the commonly used antibiotics. In the last twenty years, screening has been carried out intensively to reveal the antimicrobial activity of the Ag(I) complexes with a variety of organic substances [3-10]. Though in rare cases, microorganisms were found to be resistant to silver and its compounds. This resistance may be overcome by using silver complexes due to changes in their hydrophilic/lipophilic characteristics, solubility, structure and the ability of the complex to take part in exchange reactions with bioligands [2,11]. Thus, the more loosely bound is Ag(I) ion in coordination core of the complex, the more probable is its interaction with soft bases of target biomolecules (proteins, DNA, etc.), and primarily with sulphur-containing functional groups of proteins [12]. This concept allows one to predict the antimicrobial properties of Ag(I) complexes synthesized rather effectively. However, it doesn’t take into account the role of redox activity of Ag(I) compounds in realizing their bioactivity and doesn’t consider a possibility of correlation between these properties.

Different antibacterial agents are known to act *in vivo* as electron transfer agents in the production of radical species or disruption of normal electron transport [13]. Electron transfer can occur either to the metal center or, if in the biologically accessible range, to redox-active ligands, resulting in reactive species capable of attacking biologically relevant target molecules respectively either by ligand displacement at the metal center or by radicals formed at the ligand entity [14,15].

In our view, the redox-active Ag(I) complexes with sterically hindered phenolic derivatives are a particularly rich source of effective antibacterial agents. Nowadays phenolic derivatives as antibacterial agents are of limited utility because of their toxicity and irritating action. Introduction of substituents into benzene ring and Ag(I) ion complexation, which change the hydrophilic-lipophilic balance of a compound, will allow one not only to achieve an optimal biocidal effect but also to decrease their toxicity.

Previously we have carried out the synthesis and characterization of Ag(I) complexes with some *ortho*-diphenol derivatives (hydrazone, thiosemicarbazone derivatives, as well as derivatives of thiocarboxylic acids) [16-18]. They displayed bioactivity against microorganisms tested comparable with or even higher than that of some commonly used antibiotics and silver-containing drugs. Unfortunately, the above-mentioned Ag(I) complexes were found to be unstable in solvents with a high solvating power. They were shown to be complexes with partial charge transfer (PCT), which belong to a special class of transition metal complexes with redox-active ligands (*ortho*-dioxolenes). The intramolecular electron transfer between the catechol-containing ligand and the metal ion is observed therein [19]. Thus the Ag(I) complexes acquire partly semiquinonate character and are typified by an intramolecular redox reaction in some organic solvents resulting in the formation of silver nanoparticles (AgNPs) [20-23].

For this reason, we undertook a design of the Ag(I) complexes with phenolic Schiff bases for the purpose of inhibiting bacterial growth and improving the stability of the redox-active Ag(I) complexes in solution. We chose Schiff bases because they have important implications for drug discovery and development owing to the structural diversity and ease of synthesis; they were found to be potent against fungi, bacteria, protozoa [24]. On the other hand, the generation of AgNPs by decomposition of the Ag(I) complexes in solution deserves particular attention. We described the peculiarities of AgNPs formation on using the Ag(I) complexes with 2-[4,6-di-(*tert*-butyl)-2,3-dihydroxyphenylsulfanyl]acetic acid and 4,6-di-*tert*-butyl-2,3-dihydroxybenzaldehyde isonicotinoyl hydrazone as precursors [20-23]. According to our previous results, a positive feature of this method is the fact that the Ag(I) complex with PCT (as a precursor) includes an oxidizer, a reductant and an AgNPs stabilizer. Moreover, to understand the mechanism of activity of silver formulations, we must consider the possibility of AgNPs acting as the ultimate biocidal bullets that will contribute to the efficiency of the redox-active Ag(I) complexes [25]. Therefore, the second key task of the present work is to compare the antibacterial activity of AgNPs prepared by the above-mentioned method with that of their silver precursors.

In the earlier investigations, using cyclic voltammetry, we have shown some sterically hindered phenolic derivatives as well as their Ag(I) complexes to be also of a pronounced reducing ability correlating with antibacterial activity and the rate of the reduction of bovine heart cytochrome *c* (Fe(III)-Cyt *c*) [26]. Those results allowed us to suggest that redox processes could play an important part in the biotransformation and pharmacological activity of these compounds, and one of the possible types of their biological macromolecular targets can be components of electron transport chains such as Cyt *c*-like ones. In particular, the most sensitive enzymes’ sites for the action of silver lay between cytochrome *b* and *a3* [2]. Owing to subcellular localization, bacterial cytochromes are among the first target enzymes for anti-infective agents on their way into the cell. Account should also be taken of the known relation of the above-mentioned enzyme system to generation and detoxification of reactive oxygen species [27]. The results obtained are discussed in the view of a supposed relationship between their antibacterial activity, the capability of the compounds under study for reducing Fe(III)-Cyt c, redox properties determined by cyclic voltammetry, lipophilicity, and their ability to interact with BSA.

## Experimental

### Materials and methods

Elemental analyses were carried out with a Vario EL instrument (CHNS mode). Silver was determined using an atomic emission spectrometer with an inductively coupled plasma excitation source (Spectroflame Modula). ^1^H NMR spectra (DMSO-d_6_ was used as a solvent) were recorded using a Bruker Avance-500 spectrometer operating at 500 MHz. Mass spectra were registered with a Shimadzu QP-5000 spectrometer using direct injection of the specimens into an ion source with a temperature of the source 200 °C and ionization energy of 70 eV. UV-Vis absorption spectra of the compounds were recorded in acetonitrile (HPLC grade). Spectrophotometric experiments were performed with a Solar PB 2201 spectrophotometer using a quartz cuvette with 1 cm optical path. The IR spectra were registered with an AVATAR FT-IR-330 (Thermo Nicolet) spectrometer in the wavelength range of 400–4000 cm^–1^ using a Smart Diffuse Reflectance accessory. ESR spectra of solid samples were measured with an ERS-220 X-band spectrometer (9.45 GHz) at room temperature, using 100-kHz field modulation; *g* factors were quoted relative to the standard marker 2,2-diphenyl-1-picrylhydrazyl (DPPH). The molar conductance of 10^–4^ M solutions of the silver complexes in DMSO was measured at room temperature using a TESLA BMS91 conductometer (cell constant 1.0). Fluorometric experiments were performed with a Solar CM 2203 spectrofluorometer using a quartz cuvette with 1 cm optical path. The lipophilicity test was made by determining the *n*-octanol/water partition coefficient (logP) according to the method reported in the literature [28]. Cyclic voltammetry measurements were performed under dry nitrogen in a three-electrode two-compartment electrochemical cell using a potentiostat with glassy-carbon (GC) working electrode, Pt auxiliary electrode and Ag|AgCl|0.1 mol·l^–1^ (C_2_H_5_)_4_NCl reference electrode. The supporting electrolyte was 0.1 mol·l^–1^ (C_2_H_5_)_4_NClO_4_. The Ag|AgCl|0.1 mol·l^–1^ (C_2_H_5_)_4_NCl reference electrode was calibrated with the ferrocenium | ferrocene redox couple (this couple has potential +0.54V vs. our reference electrode). Anhydrous acetonitrile was used as a solvent. (C_2_H_5_)_4_NClO_4_ and (C_2_H_5_)_4_NCl used to prepare solutions were dried respectively at 80 and 100 °C under vacuum for 3 h. Preparation of solutions and filling the electrochemical cell were carried out in a glove box in dry nitrogen.

### Synthesis of the ligands

**Figure 1.**
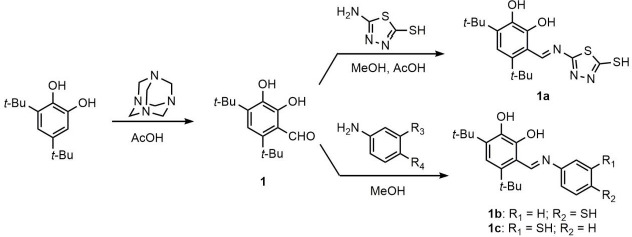
Scheme of synthesizing the ligands **1a**–**1c**.

The aldehyde **1** was obtained by Duff reaction using 3,5-di-*tert*-butylbenzene-1,2-diol and hexamethylenetetramine in glacial acetic acid as starting materials [17]. Compounds **1b**–**1c** were prepared by the reaction of aromatic amines with 4,6-di-*tert*-butyl-2,3-dihydroxybenzaldehyde **1** in the molar ratio 1:1 using anhydrous methanol as a solvent. The ligand **1a** was obtained upon refluxing aldehyde **1** with 5-amino-1,3,4-thiadiazole-2-thiol in the mixture of acetic acid and methanol (1:2) for 12 h (Fig. 1).

**Compound 1a**: Orange crystals from ethanol, yield 57 %; m.p. 111–113 °C. ^1^H NMR (500 MHz, DMSO-d_6_) δ, ppm: 1.36 s (9H, 3CH_3_), 1.44 s (9H, 3CH_3_), 6.82 s (1H, CH_Ar_), 7.09 s (1H, OH), 8.75 s (1H, CH=N), 13.17 s (1H, SH), 14.60 br. s (1H, OH). FT-IR (ν, cm^-1^): 3342s, ν(O–H); 1616s ν(C=N); 1166m, 1111m ν(C–O), 1271m ν(C_arom_–N), 2576w ν(S–H), 688w ν(C–S), 634w, 601w ν(CR–S–). UV-vis: λ, nm (logε, M^-1^ cm^-1^): 211 (4.25), 289 (4.28), 370 (3.62). Mass spectrum, *m/z* (*I*_rel_, %): 365(100) [M]^*+*^. [C_17_H_23_N_3_O_2_S_2_]^+^.

**Compound 1b**: Red solid from ethanol, yield 64 %; m.p. 189–191 °C. ^1^H NMR (500 MHz, DMSO-d_6_) δ, ppm: 1.37 s (9H, 3CH_3_), 1.44 s (9H, 3CH_3_), 5.65 br. s (1H, SH), 6.77 s (1H, CH_Ar_), 7.27–7.30 m (2H, CH_Ar_), 7.40–7.42 m (2H, CH_Ar_), 8.25 s (1H, OH), 9.36 s (1H, CH=N), 15.26 s (1H, OH). FT-IR (ν, cm^-1^): 3394s, ν(O–H); 1610s ν(C=N); 1162m, 1097m ν(C–O), 1247m ν(C_arom_–N), 2560w ν(S–H), 705w, 669w ν(C–S). UV-vis: λ, nm (logε, M^-1^ cm^-1^): 204 (4.57), 227sh, 302sh, 344 (4.45). Mass spectrum, *m/z* (*I*_rel_, %): 357(100) [M]^*+*^: [C_21_H_27_NO_2_S]^+^.

**Compound 1c**: Red solid from ethanol, yield 68 %. m.p. 138–140 °C. ^1^H NMR (500 MHz, DMSO-d_6_) δ, ppm: 1.38 s (9H, 3CH_3_), 1.45 s (9H, 3CH_3_), 5.75 br. s (1H, SH), 6.78 s (1H, CH_Ar_), 7.10 d (1H, CH_Ar_, *J* = 7.8 Hz), 7.24 d (1H, CH_Ar_, *J* = 7.8 Hz), 7.29–7.31 m (1H, CH_Ar_), 7.37 t (1H, CH_Ar_, *J* = 7.8 Hz), 8.39 s (1H, OH), 9.35 s (1H, CH=N), 15.12 s (1H, OH). FT-IR (ν, cm^-1^): 3346s, ν(O–H); 1627s ν(C=N); 1162m ν(C–O), 1263m ν(C_arom_–N), 2577w ν(S–H), 676w, 646w, 613w ν(C–S). UV-vis: λ, nm (logε, M^-1^ cm^-1^): 206 (4.43), 225 (4.36), 328 (4.27). Mass spectrum, *m/z* (*I*_rel_, %): 357(92) [M]^*+*^: [C_21_H_27_NO_2_S]^+^.

### Synthesis of the complexes

**Figure 2.**
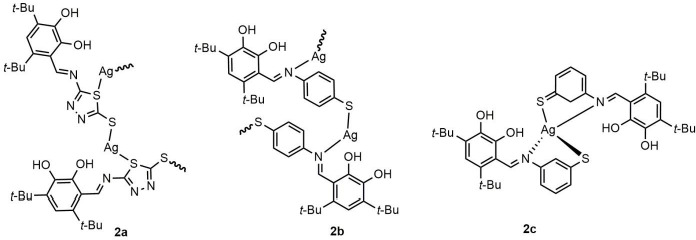
Coordination modes of the ligands **1a**–**1c** in their Ag(I) complexes **2a**–**2c**.

A novel procedure was developed for synthesizing Ag(I) complexes with the ligands **1a**–**1c** making it possible to prevent redox interaction with Ag(I) ions and to control the ligand environment of ions and thus to produce complexes with specified characteristics. The Ag(I) complexes **2a** and **2b** were isolated from the mixture of acetonitrile/acetone (1:5 v/v) upon the reaction respectively of the parent ligands **1a** and **1b** with Ag(I) nitrate in the molar ratio metal/ligand 1:1. The compound **2c** was obtained with the molar ratio metal/ligand 1:2 (Fig. 2).

**Compound 2a**: Orange powder, yield 84 %. Anal. Calcd. for C_17_H_22_AgN_3_O_2_S_2_: C, 43.23; H, 4.69; Ag, 22.84; N, 8.90; S, 13.57; Found: C, 43.14; H, 4.53; Ag, 22.91; N, 8.74; S, 13.49. ^1^H NMR (500 MHz, DMSO-d_6_) δ, ppm: 1.36 s (9H, 3CH_3_), 1.44 s (9H, 3CH_3_), 6.82 s (1H, CH_Ar_), 7.57 br. s (1H, OH), 8.75 s (1H, CH=N). FT-IR (ν, cm^-1^): 3520s, 3340s, ν(O–H); 1612s ν(C=N); 1168m ν(C–O), 1270m ν(C_arom_–N), 673w ν(C–S), 588w ν(CR–S–). UV-vis: λ, nm: 212, 288, 367. Molar conductivity Λ: 20.0 Ω^-1^cm^2^mol^-1^.

**Compound 2b**: Dark red solid, yield 95 %. Anal. Calcd. for C_21_H_26_AgNO_2_S: C, 54.32; H, 5.64; Ag, 23.23; N, 3.02; S, 6.90; Found: C, 54.26; H, 5.47; Ag, 23.19; N, 3.11; S, 6.81. FT-IR (ν, cm^-1^): 3410s, ν(O–H); 1651s ν(C=N); 1168m, 1087m ν(C–O), 1224m ν(C_arom_–N), 690w, 642w ν(C–S), 441m ν(N–Ag). UV-vis: λ, nm: 201, 288, 385. Molar conductivity Λ: 12.5 Ω^-1^cm^2^mol^-1^.

**Compound 2c**: Orange powder, yield 92 %. Anal. Calcd. for C_42_H_53_AgN_2_O_4_S_2_: C, 61.38; H, 6.50; Ag, 13.12; N, 3.41; S, 7.80; Found: C, 61.21; H, 6.01; Ag, 13.34; N, 3.32; S, 7.74. ^1^H NMR (500 MHz, DMSO-d_6_) 1.25 s (18H, 3CH_3_), 1.32 s (18H, 3CH_3_), 6.65 s (2H, CH_Ar_), 6.78 br. s (5H, CH_Ar_), 7.27 br. s (2H, CH_Ar_), 7.43 br. s (2H, CH_Ar_), 8.11 s (2H, OH), 9.16 s (2H, CH=N), 15.11 br. s (2H, OH). FT-IR (ν, cm^-1^): 3504s, ν(O–H); 1610s ν(C=N); 1162m ν(C–O), 1245m ν(C_arom_–N), 632w, 572w ν(C–S); 1072s ν(C=S), 443m, 418m ν(N–Ag). UV-vis: λ, nm: 209, 291, 385. Molar conductivity Λ: 3.7 Ω^-1^cm^2^mol^-1^.

### Synthesis of silver nanoparticles (AgNPs)

**Figure 3.**
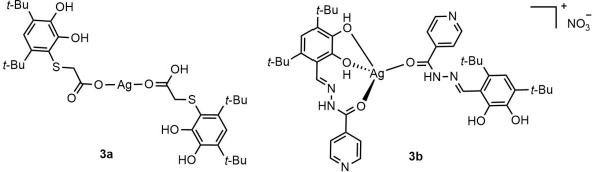
Plausible structures of the Ag(I) complexes **3a** and **3b**.

Silver organosols were produced by chemical decomposition of the Ag(I) complexes with 2-[4,6-di-(*tert*-butyl)-2,3-dihydroxyphenylsulfanyl]acetic acid (**3a**) and 4,6-di-*tert*-butyl-2,3-dihydroxybenzaldehyde isonicotinoyl hydrazone (**3b**) (Fig. 3) when the latter was dissolved in media with high donor numbers *DN* > 19 (ethanol, propanol-2, butanol-1, dimethyl formamide, dimethyl sulphoxide [29]) under permanent stirring. Synthesis and characterization of the Ag(I) complexes **3a** and **3b** used in this work were described elsewhere [16-18]. These sols were produced from such an amount of the complex dissolved in an organic solvent as to provide the silver concentration in the sol equal to 10^-4^ M. The sols were stored in closed, light-tight vessels. If required, argon was bubbled through the solutions to ensure the absence of oxygen. AgNPs in solid-state were prepared by evaporating the solvent using a rotary evaporator RV 8 V (IKA). Measuring the mass of the dry residue, the silver concentration in the sol was found to be 88.9–90.0 μg·ml^–1^ [20,22,23]. The silver content in the solid phase was determined using atomic emission spectrometry and was equal to 12.1 % [20,22,23].

### Antibacterial activity

The following test microorganisms (collection of Department of Molecular Biology, Belarusian State University) were used: Gram-negative bacteria (*Escherichia coli*, *Pseudomonas putida*, *Proteus vulgaris, Pantoea agglomerans, Serratia marcescens, Pectobacterium carotovorum*, *Salmonella typhimurium*, *Pseudomonas aeruginosa*), Gram-positive bacteria (*Bacillus subtilis*, *Staphylococcus saprophyticus*, *Bacillus pumilus P10*, *Bacillus pumilus B9*, *Staphylococcus aureus, Sarcina lutea*). The microorganisms were subcultured for testing in Muller-Hinton broth (Merck) (pH 7.4±0.2). The cells were suspended, according to the McFarland protocol, in saline solution to produce a suspension of about 10^6^ CFU/ml (colony-forming units per ml). Because all the compounds tested are almost insoluble in water, they were dissolved, according to standard recommendations [30,31], in a small volume of an organic solvent (dimethyl sulfoxide), which is well miscible with the aqueous nutrient medium on dilution and doesn’t react with the compounds tested. If organic solvent was diluted in Mueller-Hinton broth at 1:10 to 1:100, there was no detectable inhibition or antagonism associated with the residual solvent concentration when tested against bacteria [30,31]. This solvent is used for making stock solutions (1.6 mg·ml^–1^) of the test compounds. Stock solutions can be further diluted with the broth. Serial dilutions of stock solutions to certain concentrations were carried out in test tubes. Concentrations of the compounds under study evaluated in Mueller-Hinton broth ranged from 400 μg·ml^–1^ to 1.6 μg·ml^–1^. A specified volume of a 24 h old inoculum was added to each tube. The organic solvent (dimethyl sulfoxide) at the final concentration (1–2 %) in the nutrient medium did not affect the growth of the test microorganisms. The incubation was carried out at the optimal growth temperature for 24 h, and optical density (OD_600_) was determined for the bacterial cultures with and without the compounds tested. The contents of the test tubes in which the concentration of these compounds was sufficient to suppress the microbial growth remained clear, while their turbidity was evidence for the presence of bacteria. The MIC, defined as the lowest concentration of the test compound that inhibits the visible microbial growth, was determined after incubation. MIC values are given in μmol·ml^–1^ to reveal a correlation between the antibacterial activity and reducing ability of the compounds. Tests using dimethyl sulfoxide as negative control were carried out in parallel. Results were always verified in three separate experiments.

### Interaction with BSA

In the fluorescence quenching experiment, quenching of the tryptophan residues of BSA was done by keeping the constant concentration of BSA (2 μM) in 5 mM Tris-HCl buffer (pH 7.4) with 50 mM NaCl while varying that of the complexes (0–10 μM). The fluorescence spectra were recorded in the range of 310–450 nm upon excitation at 295 nm and an emission wavelength of tryptophan residues of BSA at 339 nm after each addition of the quencher. Solutions of the complexes were prepared in DMSO. All experiments were carried out at room temperature. The fluorescence quenching is described by the Stern-Volmer equation [32]:


(1)

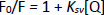



F and F_0_ are fluorescence intensities respectively with and without the quencher (the complex), *K*_sv_ is a Stern–Volmer quenching constant, and [Q] is the quencher concentration. The quenching constant (*K_sv_*) was calculated from the slope of the plot of F_0_/F versus [Q]. The Hill coefficient (*n*) and the apparent binding constant (*K_b_*) values were calculated from the plot of log [(F_0_ – F)/F] versus log[Q] according to the following equation [32]:


(2)

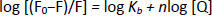



### Interaction with cytochrome c

Bovine heart Fe(III)-Cyt *c* (Sigma) was used. Fe(III)-Cyt *c* concentration was determined on its interaction with excess sodium dithionite using the absorption coefficient ε_550_ = 21000 M^-1^cm^-1^ [33,34]. Argon-saturated DMSO solutions of the ligands and complexes and Fe(III)-Cyt *c* (7 μM) were used. Experiments were performed in 10 mM Tris-HCl buffer (pH 7.6) at 20 °C. Aliquots of the compounds under study were added to Fe(III)-Cyt *c* solution up to the final concentration 35.0 μM. The initial rate of Fe(III)-Cyt *c* reduction (ν_0_) was calculated by the slope of the linear portion of kinetic curve A_550_ versus time. The results were confirmed in three independent experiments.

## Results and Discussion

### Physicochemical characterization

The catechol-based ligands **1a**–**1c** were obtained via condensation of a primary amine with aldehyde **1** (Fig. 1). The Ag(I) complexes **2a**–**2c** were isolated in amorphous or poorly crystalline state from the mixture of acetone/acetonitrile. They were practically insoluble in non-polar organic solvents, slightly soluble in acetonitrile, but soluble in DMSO and DMF. The values of the molar conductivity (Λ ≤ 20 Ω^-1^cm^2^M^-1^) in DMSO characteristic of the metal complexes under study indicate that they are non-electrolytes [35].

^1^H NMR spectra of the ligands and the Ag(I) complexes were recorded in DMSO-d_6_. The spectra of Schiff bases **1a**–**1c** display two singlets at 7.09–8.39 and 14.60–15.26 ppm corresponding to the phenolic protons of a catechol moiety. The aromatic protons of the catechol moiety were observed as singlets at ~ 6.8 ppm. The formation of the ligands **1a**–**1c** was also evidenced by the appearance of singlets at 8.75–9.36 ppm corresponding to the azomethine protons. The signals of SH protons resonated at 5.65, 5.75 and 13.17 ppm as singlets. The disappearance of these signals in the spectra of Ag(I) complexes **2a** and **2c** indicates the participation of the mercapto group in the coordination via deprotonation. The signal of CH=N proton shifted and was observed at 9.16 ppm for **2c**, indicating the involvement of the azomethine group in Ag(I) coordination. However, in the spectrum of **2a** this signal virtually did not change its position in the spectrum. The minor shift of the hydroxyl proton signals in the spectra of the complexes may result from the participation of hydroxyl groups in different processes: coordination of these groups to the metal ion as well as disruption of the strong hydrogen bonding between the free ligand molecules and the formation of hydrogen bonding involving two uncoordinated hydroxyl groups of one of the ligands.

Analysis of IR spectra of **1a**>**1c** and their Ag(I) complexes made it possible to identify donor sites of binding of the ligands to Ag(I). The general characteristic of IR spectra of these complexes is the fixed position of the bands corresponding to vibrations of the structural fragments not bonded to Ag(I) (benzene ring, *tert*-butyl groups), which are present in the spectra of the free ligands [36,37]. It should be emphasized that the band characteristic of *o*-benzoquinones [38] is missing from the spectra of the complexes, thus suggesting the lack of any oxidized ligands in the coordination sphere of the latter. In the spectra of the ligands **1a**>**1c** in the region 3394–3342 cm^–1^ there is a broad band assigned to the stretching vibrations of phenolic O–H groups, and in the spectra of the Ag(I) complexes, it is blue-shifted, suggesting that there is no coordination of oxygen atoms to Ag(I). This fact is also supported by the lack of changes in the range of 1200–1100 cm^–1^ corresponding to the stretching C–O bond vibrations. In the spectra of the complexes **2b** and **2c**, there is a shift of the bands in the region 1651–1610 cm^–1^ corresponding to the stretching C=N bond vibrations. Besides, new bands appearing in the region 500–400 cm^–1^ suggest nitrogen atom participating in coordination to Ag(I) ion. By contrast, new bands missing from the range of 500–400 cm^–1^ and no changes in the position and intensity of the bands in the spectrum of the complex **2a** assigned to the stretching C=N bond vibrations (1616–1610 cm^–1^) suggest that nitrogen atoms take no part in the formation of coordination core of the complex. In the spectra of the ligands **1a**>**1c** in the region 2600–2500 cm^–1^ there is a weak band assigned to the stretching S–H bond vibrations, absent in the spectra of all the complexes, which is evidence for S atom coordinating in thiolate form. In the spectra of the complexes **2a** and **2b** there are no bands that could be assigned to the stretching C=S bond vibrations (1050–1200 cm^–1^). But there is a very strong band at 1072 cm^–1^ in the spectrum of the complex **2c**, suggesting that S atom is coordinated in thion-form along with thiolate one. In the complex **2a** it is the sulphur atom of the heterocycle which takes part in coordination to Ag(I), as is evidenced by the red shift of the band belonging to the stretching –CR–S– bond vibrations (the latter is present at 630–600 cm^–1^ for the ligand **1a**).

The UV-vis absorption spectra of the ligands exhibit bands in the range of 204–227 nm, which may be assigned to π-π* transitions of the aromatic rings [39]. The intense bands at 289–370 nm are due to π-π* and *n*-π* transitions of the azomethine group [39]. In the spectra of the complexes **2b** and **2c**, these bands are shifted to the region 388–385 nm, indicating the participation of azomethine group in the coordination. The bands in the spectrum of **2a** virtually do not change their position.

As the compounds **1a**>**1c** are potential Ag(I) reductants, their complexes **2a**–**2c** were investigated by ESR method in order to elucidate the possibility of the formation of paramagnetic particles by virtue of redox interaction between Ag(I) ions and phenolic ligands. ESR method is known to be widely applied to detect and identify paramagnetic silver species (Ag(0) and Ag(II)) [40]. However, it was found that they were absent in the Ag(I) complexes **2a**–**2c**, as no their characteristics (*g*-factors) were registered in the ESR spectra.

For interpretation of the ESR spectra of these Ag(I) complexes, a concept can be used of complexes with PCT as a special class of compounds of transition metal ions with redox ligands [19]. Their peculiarity is the existence of rapidly establishing thermal equilibrium between the ground and electron-excited states. Owing to this, a PCT complex can interact with any “third” particle, being simultaneously in two (ground and electron-excited) states, and can demonstrate properties both of initial reacting particles and of electron-transfer products. “Ambiguity” of properties of PCT complexes is clearly interpreted in terms of the Mulliken's theory [41], according to which a PCT complex may be conceived as a superposition of states without charge transfer and with a complete charge transfer. The PCT complex decomposes from its ground state to yield the initial reagents, while on decomposing from an electron-excited one, it gives electron-transfer products. Thus, according to our previous results, the redox-active Ag(I) complexes **3a** and **3b** with sterically hindered *o*-diphenol derivatives (Fig. 3) were found to acquire partly semiquinonate character (*g*_iso_=2.003÷2.004), and on dissolving in solvents with high donor numbers (DN > 20) that actively solvate metal cations the PCT can initiate a redox process resulting in, as noted above, colloidal silver formation [20-23]. In contrast, the behaviour of the complexes **2a**–**2c** is different from that previously reported for other redox-active Ag(I) complexes [16,18,21]. It should be emphasized that no singlet signal with *g*_iso_ close to *g_e_* is registered in the ESR spectra of the complexes **2a**–**2c**. Besides, on dissolving these novel Ag(I) complexes in strongly solvating solvents, no AgNPs are formed. The results obtained support the absence of the PCT in complexes **2a**–**2c**. Thus, the results of the spectral studies suggest an asymmetric mode of binding to functional groups involved in complexation, and the general mode of ligating atoms in the Ag(I) complexes **2a**–**2c** can be represented as shown in Fig. 2.

### Redox characterization: electrochemical study and interaction with cytochrome c

The redox properties of the newly synthesized compounds **1a**–**1c** and their Ag(I) complexes **2a**–**2c** were determined electrochemically using cyclic voltammetry method. The latter is the most widely used technique for acquiring qualitative information about electrochemical reactions; it offers a rapid location of redox potentials of the electroactive species [42]. Cyclic voltammetry allows a successful evaluation of the total antioxidant capacity because the redox potentials of phenolic compounds determined by this method are considered good measures of their reducing ability [43,44].

There are essential reasons for carrying out an investigation of the redox properties of phenolic ligands and their Ag(I) complexes. First, the phenolic ligands in metal complexes can be in different redox states depending on conditions (diamagnetic single- or double-charged anions, neutral *ortho*-benzoquinones or paramagnetic *ortho*-benzosemiquinone anion-radicals [45]. Second, according to our previous data [21,26], it is safe to assume that the redox properties of the phenolic ligands and their Ag(I) complexes can affect their bioactivity. It was found that the redox processes involving the compounds under study are reversible or irreversible. That is why the potential value for the first oxidation peak (E_pa_^1^, V) was taken as a criterion to compare the reducing ability of these compounds of the same type, and the more cathodic this value is, the more active is the compound as a reductant [42]. On the basis of the electrochemical findings, the compounds investigated can be graded in their reducing ability as follows (Table 1): **1a**>**1c**≈**1b**; **2a**>**2b**>**2c.** Since the Ag(I) ion in the complexes does not undergo any electrochemical transformations, the anodic processes can be assigned to ligand-centered ones. The oxidation potential ranges of the ligands are close. Furthermore, it was found that the complexes rank below the ligands in reducing ability.

We have carried out a spectrophotometrical investigation of bovine heart Fe(III)-Cyt *c* reduction with **1a**–**1c** and their Ag(I) complexes **2a**–**2c**. It was established that among the compounds under study it is the ligands **1b** and **1c** that are characterized by the highest rate of Fe(III)-Cyt *c* reduction (Table 1), and they show a higher reducing ability (determined electrochemically) as compared with that of their Ag(I) complexes **2b** and **2c**. Among the Ag(I) complexes, it is **2a** that demonstrates the highest level of Fe(III)-Cyt *c* reduction rate, and according to electrochemical data, this complex is the most active reducing agent. On the contrary, the complex **2a** is a weaker reductant than the respective ligand **1a**, but the latter reduces the enzyme slower than **2a.** Thus, the reduction of Fe(III)-Cyt *c* with the sterically hindered phenolic derivatives and their Ag(I) complexes cannot be due solely to their capacity for oxidation, depending on other physicochemical properties (ionization, solubility etc.) in a more intricate way.

### Antibacterial activity

Increased activity of Ag(I) complexes compared to silver nitrate is known [2]. This effect can be associated with an increase in compound lipophilicity owing to Ag(I) complexation with organic ligands. A microbiological investigation of the newly synthesized compounds **1a**–**1c** and their Ag(I) complexes **2a**–**2c** was carried out *in vitro* for test cultures of Gram-positive and Gram-negative bacteria using the procedures described in *Section* “*Antibacterial activity”*; the data obtained are given in Tables 2 and 3. Microbiological tests show that the Ag(I) complexes are more active than the parent ligands. The Ag(I) complexes suppress the growth of test cultures at moderate concentrations, and the activity of the Ag(I) complex **2c** exceeds not only the inhibiting action of the ligands but also that of other Ag(I) complexes, **2b** and **2a** (Table 2). We have chosen thiol-containing Schiff bases **1a**–**1c** as the ligands for improving the stability of the redox-active Ag(I) complexes in solution due to the high affinity of Ag(I) ions for the thiolate sulphur and the high stability of the Ag(I) complexes with organosulphur compounds (*K* ~ 10^13^) [46]. However, when evaluating the complexation effect of these ligands with Ag(I) ions on their antibacterial properties, one is forced to accept the fact that the newly synthesized Ag(I) complexes **2a**–**2c** are characterized by MIC values higher than those of the Ag(I) complexes **3a** and **3b** with sterically hindered phenolic derivatives previously reported by us [17,21] (Table 3).

Based on the literature and our experimental data, several explanations for the effect observed can be offered. First, the complex-forming Ag(I) ion is bonded to sulphur atom in coordination cores of the Ag(I) complexes and thus, according to [2,12], is not able to take part in exchange reactions with bioligands, interacting with soft bases (nitrogen or sulfur atoms) in the composition of target biomolecules of a microbial cell. Second, the Ag(I) complexes **3a** and **3b** are effective antioxidants (unlike **2a**–**2c**) owing to a special state of silver in their molecules resulting from PCT complex being formed, and for redox-active complexes, which are active reductants, the antimicrobial activity can be related to their action on electron-transport systems of a microbial cell [21,26,47].

The toxic effect is believed to result generally from the catalytic production of reactive radical species that arise *via* electron transfer and destroy the cell. Third, on carrying out microbiological assays in a liquid nutrient medium, it is necessary to take into account the possibility of silver nanoparticles being formed as a result of the PCT complex decomposition [20-23]. The mean size of these particles is 5 nm, and it is known that silver particles of this very size demonstrate the highest antimicrobial activity [25,48,49].

In our earlier investigations, we have shown that the Ag(I) complexes **3a** and **3b** are the AgNPs precursors. In this paper, we investigated the antibacterial activity of AgNPs produced by decomposition of the Ag(I) complexes **3a** and **3b** (respectively AgNPs^3a^ and AgNPs^3b^, see *Section “Synthesis of silver nanoparticles”*). The data obtained are given in Table 3. AgNPs^3a^ and AgNPs^3b^ as well as the Ag(I) complexes **3a** and **3b** are characterized by very low MIC values, their antimicrobial activity exceeds that of the commonly used antibiotics (Table 3).

### Interaction with BSA

**Figure 4.**
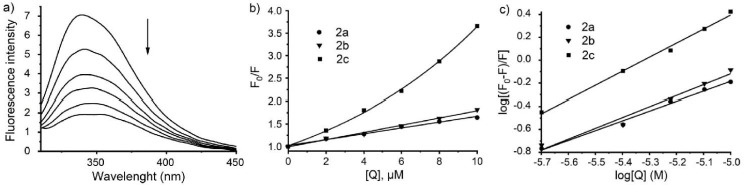
a) Fluorescence spectra of BSA without the complex **2c** and with it at different concentrations. b) Stern-Volmer plot for fluorescence quenching of BSA by the complexes **2a**–**2c**. c) Double logarithmic plot log[(F_0_-F)/F] *vs* log[Q].

Serum albumins are the most abundant proteins in the circulatory system of mammals, responsible for the maintenance of oncotic pressure and blood pH. They also serve as depot and transport proteins for a variety of endogenous and exogenous substances such as drugs, hormones, fatty acids and metal ions [50]. It is known that the affinity of a drug for plasma proteins influences its distribution, efficacy, free concentration, and metabolism. Therefore, investigation of protein-drug interactions is important. Recently it was found that BSA interacted with Ag(I) ions, forming a protein-silver adduct. Quenching the fluorescence of BSA with AgNPs was also studied [50]. In this work, the distinguishing features of the interaction between Ag(I) complexes **2a**–**2c** and BSA were determined using fluorescence quenching experiments. BSA was chosen as a model protein in biochemical studies due to its structural homology with human serum albumin [51]. BSA is composed of three domains (I, II and III) divided into two subdomains (A and B), and contains two tryptophan residues, Trp-134 and Trp-213, which can be used as fluorophores. Upon addition of the Ag(I) complexes to BSA solution, there was a decrease in fluorescence (Fig. 4, a). Fluorescence data were analyzed to estimate the binding constants of the Ag(I) complexes to BSA according to a 1:1 model. Stern-Volmer constant (*K_sv_*) was calculated from the slope of the plot of F_0_/F versus [Q] (Equation (1)). The apparent binding constant (*K_b_*) and Hill coefficient (*n*) were calculated according to Equation (2). The data obtained are presented in Table 4. In the case of complex **2c** there is a positive deviation from the linearity of the Stern-Volmer plot (Fig. 4, b), which occurs primarily either when the extent of quenching is large or due to the combination of the static and dynamic mechanisms of quenching.

To evaluate *K_sv_*, we considered the range within which the Stern-Volmer plot was linear. According to the results obtained, complex **2c** demonstrated a high affinity for the BSA molecule with log*K_b_* = 6.5 M^–1^, while log *K_b_* values of the polymeric complexes **2a** and **2b** were much lower. The Hill coefficient for **2c** was greater than unity, indicating positive cooperativity. The *n* values calculated for **2a** and **2b** were lower than unity, suggesting negative cooperativity. Binding constant decreased in the order: **2c**<**2b**<**2a**.

It also should be noted that the direct correlation between the binding constant and log *P* was observed in the series of Ag(I) complexes. The log *K_b_* for these complexes was significantly lower than AgNPs reported previously [50].

## Conclusions

The novel Ag(I) complexes **2a**–**2c** with sterically hindered phenolic Schiff bases were synthesized and isolated in the amorphous state. According to the data obtained, they are characterized by distorted geometry of the coordination cores [AgN_2_S_2_], [AgNS] and [AgS_2_]. The results of our investigation show that structural modification of the phenolic ligands and complexation with Ag(I) ions provides a way of obtaining Ag(I) complexes which are stable in organic solvents with high solvating power, being the complexes without PCT. The complexes **2a**–**2c** are not typified by the intramolecular redox reaction in organic solvents resulting in the formation of AgNPs. This property makes them different from the Ag(I) complexes of 2-[4,6-di(*tert*-butyl)-2,3-dihydroxyphenylsulfanyl]acetic acid and 4,6-di-*tert*-butyl-2,3-dihydroxybenzaldehyde isonicotinoyl hydrazone with PCT (**3a** and **3b)**.

The novel Ag(I) complexes with Schiff bases as well as AgNPs^3a^ and AgNPs^3b^ have been screened for their activity against different species of bacteria. All the complexes are more active than the respective ligands **1a**–**1c**, but significantly less active than the complexes **3a**, **3b**, AgNPs^3a^ and AgNPs^3b^. This antibacterial effect is believed to result generally from the possibility of the PCT complex decomposition on carrying out microbiological assays in liquid nutrients to give AgNPs. Compared with Ag(I) complexes, the mechanism for the antimicrobial action of AgNPs may be similar, but AgNPs may have much better efficiency owing to their surface area to volume ratio is higher [48,52]. In particular, AgNPs or Ag(I) ions can attack the respiratory chain in bacterial mitochondria and lead to cell death [53]. It was found that the ligands **1b**, **1c** and the complex **2a** with a high reducing ability (determined electrochemically) are characterized by the highest rates of Fe(III)-Cyt *c* reduction among the compounds synthesized. But the level of their antibacterial activity was shown to be independent of their reducing ability (determined electrochemically). Thus, further studies are required to elucidate the mechanism of the effects observed because no “one-to-one” correspondence between their bioactivity and reducing ability was established. In this paper, we presented the results characterizing the peculiarities of the interaction of the Ag(I) complexes with BSA. These results may be useful in understanding how Ag(I) compounds convert to toxic forms and will provide a pharmacological basis for novel antimicrobial agents.



## Figures and Tables

**Table 1.** Voltammetry data and rates of reduction of Fe(III)-Cyt *c* for the compounds **1a**–**1c** and their Ag(I) complexes **2a**–**2c** (anodic polarization).

Compounds	*E*^1^_pa_, V	ν_0_ (nmol min^-1^)
**1a**	0.90	0.7
**1b**	1.04	4.2
**1c**	1.02	3.9
**2a**	0.96	1.1
**2b**	1.26	0.4
**2c**	1.34	0.6

**Table 2.** Antibacterial activity of the compounds **1a**–**1c** and their Ag(I) complexes **2a**–**2c**.

Bacterial strain	Minimal inhibitory concentration (μmol·ml^–1^)					
	1a	1b	1c	2a*	2b*	2c
*Bacillus subtilis*	>0.274	0.280	0.280	0.106	0.108	0.061
*Staphylococcus saprophyticus*	>0.274	0.280	>0.280	0.106	0.108	0.061
*Proteus vulgaris*	>0.274	>0.280	>0.280	0.106	0.108	0.061
*Pantoea agglomerans*	>0.274	>0.280	>0.280	0.106	0.108	0.061
*Pseudomonas putida*	>0.274	>0.280	>0.280	0.106	0.108	0.061
*Escherichia coli*	>0.274	0.280	0.280	0.106	0.108	0.061
*Pectobacterium carotovorum*	>0.274	>0.280	>0.280	>0.106	>0.108	>0.061
*Serratia marcescens*	0.274	>0.280	>0.280	0.106	0.108	0.061
*Bacillus pumilus P10*	>0.274	>0.280	>0.280	>0.106	>0.108	>0.061
*Bacillus pumilus B9*	>0.274	>0.280	>0.280	0.106	0.108	0.061

*MIC was calculated per one monomeric unit

**Table 3.** Antibacterial activity of AgNPs, the Ag(I) complexes **3a**, **3b**.

Bacterial strain	Minimal inhibitory concentration (μmol·ml^–1^)						
	3a	3b	AgNPs^3a^	AgNPs^3b^	STM*	TCN*	CHL***
*Bacillus subtilis*	<0.004	0.004	0.007	0.007	0.011	0.014	0.009
*Staphylococcus saprophyticus*	<0.004	<0.004	0.007	0.007	0.011	0.014	0.019
*Serratia marcescens*	<0.004	<0.004	0.007	0.007	0.011	NA	0.019
*Sarcina lutea*	<0.004	<0.004	0.007	0.007	0.021	0.014	NA
*Pseudomonas aeruginosa*	<0.004	<0.004	0.007	0.007	0.172	0.056	0.039
*Escherichia coli*	<0.004	<0.004	0.007	0.007	0.005	0.007	0.019
*Salmonella typhimurium*	<0.004	<0.004	0.007	0.007	0.021	0.014	0.019
*Staphylococcus aureus*	<0.004	0.004	0.007	0.007	0.011	0.007	0.019

*streptomycin

**tetracycline

***chloramphenicol

**Table 4.** Stern-Volmer constants, apparent binding constants, Hill coefficients and log*P* of the Ag(I) complexes-BSA interactions.

Compound	*K_sv_* 10^-4^ (M^-1^)	log *K_b_* (M^-1^)	*n*	log *P*
**2a**	6.48±0.03	4.08±0.23	0.85	2.5
**2b**	7.99±0.03	4.59±0.46	0.94	2.8
**2c**	20.5±0.5	6.53±0.28	1.23	2.9
