# Supplementary material for: Silver(I) complexes with phenolic Schiff bases: Synthesis, antibacterial evaluation and interaction with biomolecules
Source: ADMET DMPK. 2022 Sep 13;10(3):197–212. doi: 10.5599/admet.1167 (PMC9484703; doi:10.5599/admet.1167)
Supplement: Supplementary file 1 [file Admet-10-1167_S1.pdf]

ADMET & DMPK X(Y) (20xx) pp-pp; doi: https://doi.org/10.5599/admet.1167

Open Access : ISSN : 1848-7718

http://www.pub.iapchem.org/ojs/index.php/admet/index

Supporting Information

Silver(I) complexes with phenolic Schiff bases: Synthesis, antibacterial evaluation and interaction with biomolecules

Natalia Loginova, Maxim Gvozdev, Nikolai Osipovich, Alina Khodosovskaya, Tatiana Koval'chuk-Rabchinskaya, Galina Ksendzova, Dzmitry Kotsikau and Anatoly Evtushenkov

UV-Vis spectra

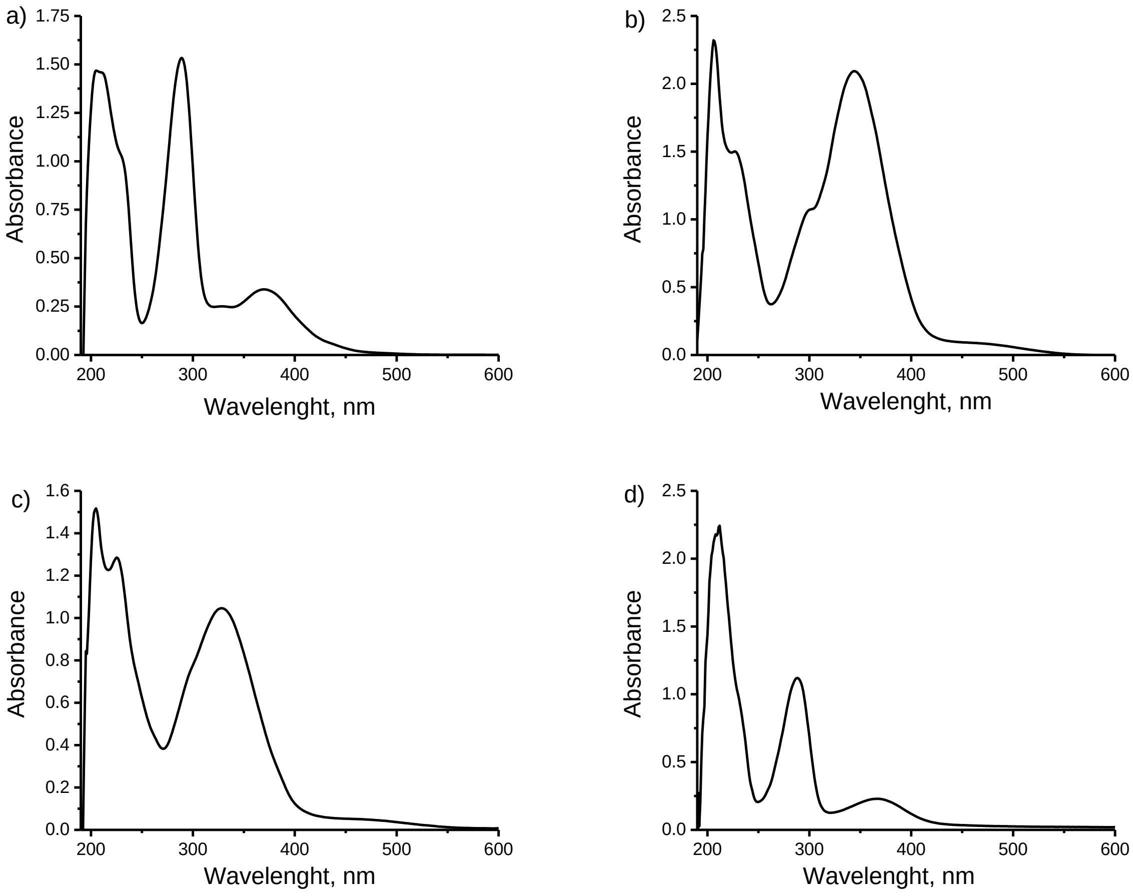

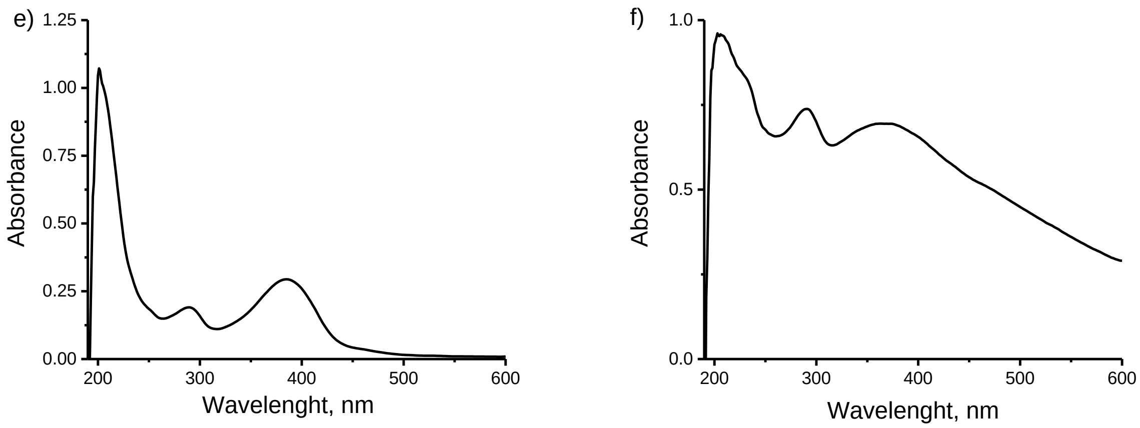

Figure S1. UV-vis spectra of the ligands **1a** (a), **1b** (b) **1c** (c) and Ag(I) complexes **2a** (d), **2b** (e), **2c** (f).

Cyclic voltammetry

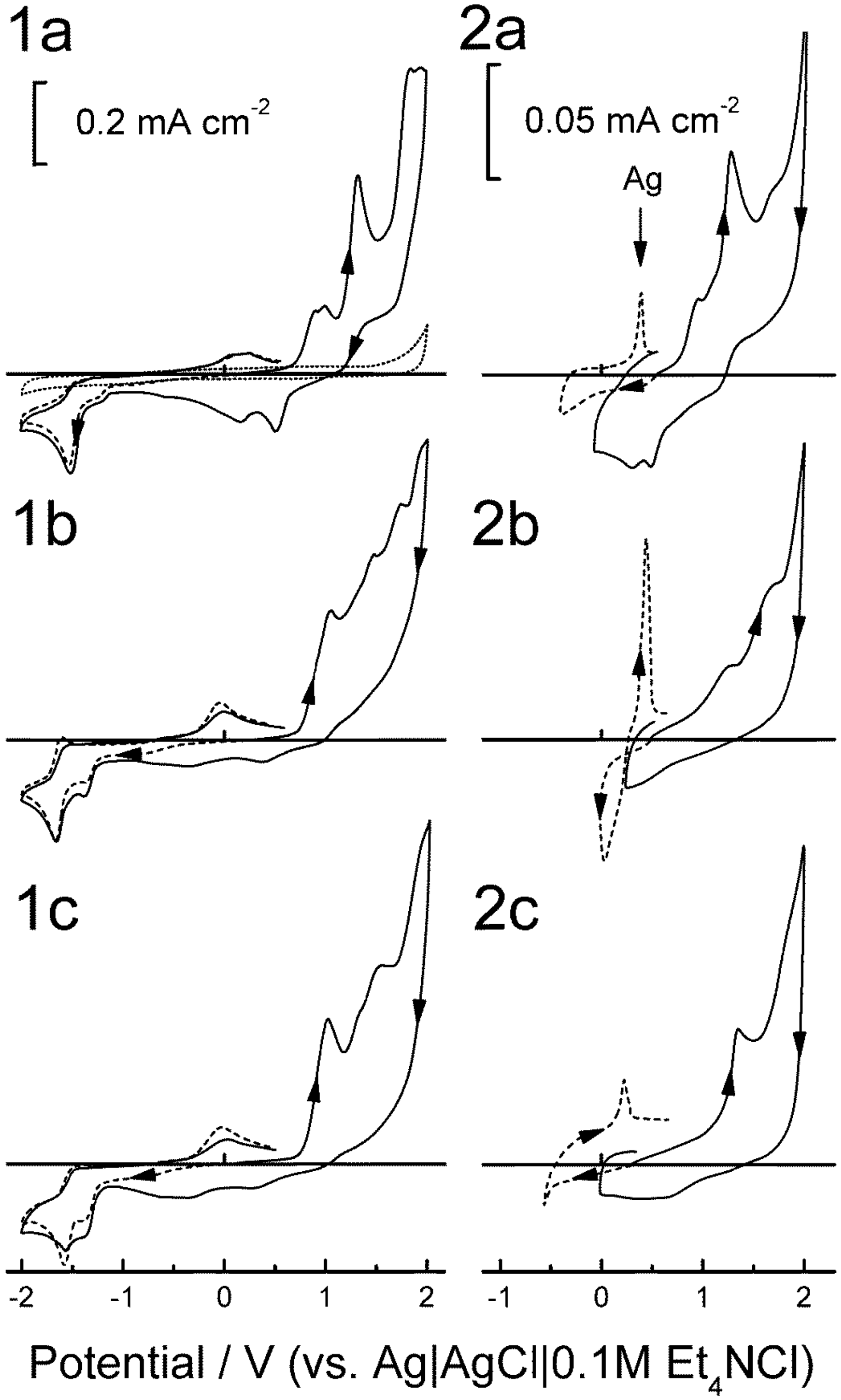

Figure S2. Voltammograms (50 mV s^{-1}) of the compounds **1a**, **1b** and **1c** (1.36 mmol $l^{-1)}$ and their Ag(I) complexes **2a**, **2b** and **2c** (0.68 mmol l^{-1}) in 0.1 mol l^{-1} $(C_2H_5)_4NClO_4$ acetonitrile solution under dry nitrogen on glassy-carbon working electrode (dotted line – background cyclic voltammogram of glassy-carbon electrode). Solid line – polarization in the anodic direction from the open circuit potential; dashed line – polarization in the cathodic direction from the open circuit potential.

Note: The compounds **1a**, **1b** and **1c** have several oxidizable functional groups (Fig. 1), and upon anodic polarization 4–5 oxidation peaks in the range 0.7–2.0 V are present in the voltammograms. Upon cathodic

polarization, it is only the reduction of the azomethine group that is observed, two cathodic peaks in the range –1.2 ÷ –2.0 V corresponding to this process. For the Ag(I) complexes **2a**, **2b** and **2c**, the participation of the oxidizable functional groups in complexation (Fig. 2) results in a decrease in the number of oxidation peaks upon anodic polarization. Upon cathodic polarization the reduction of silver is observed at potentials below zero (the sharp peak of its oxidation upon the reverse scan is marked by the arrow in Fig. S2).

Interaction with Cyt *c*

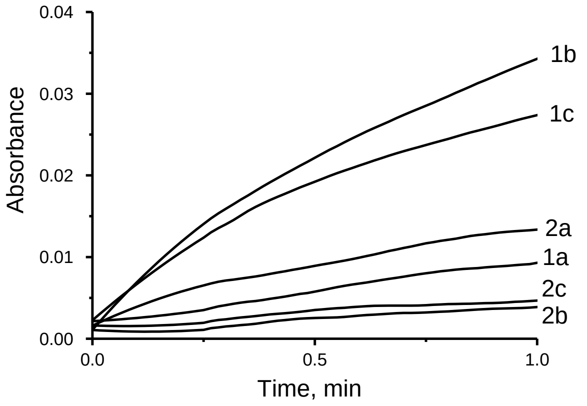

Figure S3. Kinetic curves A *vs* time for compounds under study (final concentration of Cyt *c* 7 μM).

ESP study

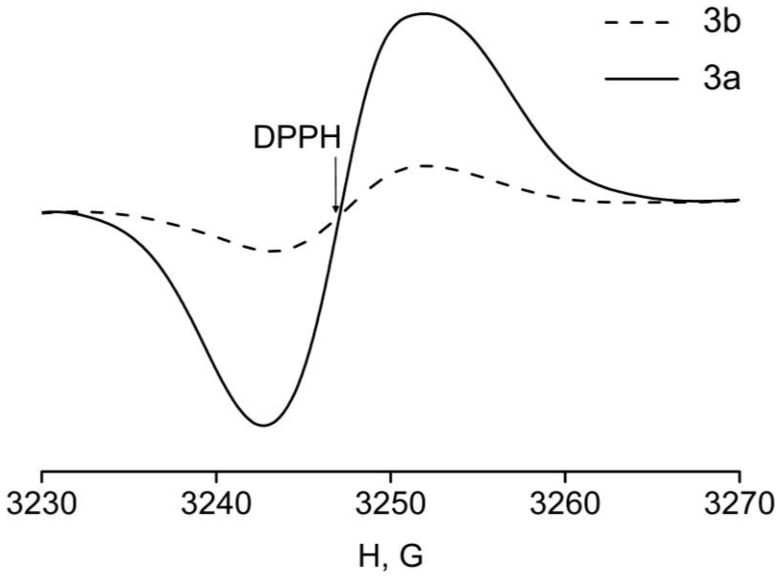

Figure S4. The ESR spectra of the Ag(I) complexes in solid state.
